# Beta-Elemene Reduces the Malignancy of Non-Small Cell Lung Cancer by Enhancing *C3orf21* Expression

**DOI:** 10.3389/fonc.2021.571476

**Published:** 2021-05-07

**Authors:** Hu Cai, Lili Ren, Ying Wang, Yongjun Zhang

**Affiliations:** ^1^ Department of Integration of Traditional Chinese and Western Medicine, Cancer Hospital of the University of Chinese Academy of Sciences (Zhejiang Cancer Hospital), Institute of Basic Medicine and Cancer (IBMC), Chinese Academy of Sciences, Hangzhou, China; ^2^ Department of Gynecological Oncology, Cancer Hospital of the University of Chinese Academy of Sciences (Zhejiang Cancer Hospital), Institute of Basic Medicine and Cancer (IBMC), Chinese Academy of Sciences, Hangzhou, China

**Keywords:** elemene injection, C3orf21, non-small cell lung cancer, malignancy, mechanisms

## Abstract

**Background:**

Beta-elemene has potent anti-tumor effect, but its anti-tumor mechanism remains unclear. Chromosome 3 open reading frame 21 (C3orf21) acts as a tumor suppressor. This study tested whether the anti-tumor effect of beta-elemene was associated with modulating C3orf21 expression in non-small cell lung cancer (NSCLC).

**Materials and Methods:**

The impact of beta-elemene on C3orf21 expression in NSCLC cells was quantified. The stable C3orf21 silencing A549 and over-expressing PC-9 cells were established and their effects on the beta-elemene-attenuated proliferation, wound healing and invasion of NSCLC cells as well as the expression of key regulators and signal events were determined.

**Results:**

Beta-elemene significantly up-regulated C3orf21 expression in NSCLC cells. Beta-elemene treatment significantly attenuated the proliferation, wound healing and invasion of NSCLC cells, which were significantly mitigated by C3orf21 silencing, but enhanced by C3orf21 over-expression. Similar patterns of beta-elemene-modulated cyclinD1, c-Myc, COX2, MMP2, MMP9, VEGF, PTEN and Notch1 expression were detected in NSCLC cells.

**Conclusions:**

Such data indicated that beta-elemene treatment attenuated the malignancy of NSCLC cells by up-regulating C3orf21 expression. Our findings may provide new mechanisms underlying the pharmacological action of beta-elemene.

## Introduction

Non-small cell lung cancer (NSCLC) is the most prevalent malignant lung tumors with high cancer-related mortality worldwide (1, 2). At present, the major treatments for NSCLC are surgical resection, target therapies and chemotherapy. Although these therapeutic strategies for NSCLC have greatly prolonged the survival of patients, many NSCLC patients commonly develop resistance to target therapies (3). This, together with the fact that many NSCLC patients are diagnosed at advanced stages, leads to a low rate of five-year survival (4). Hence, discovery of new therapeutic reagents and their acting mechanisms will be of high significance in management of NSCLC patients.

Beta-elemene, a natural lipid-soluble plant drug, can be extracted from traditional Chinese medicine *Rhizoma zedoariaem* (5, 6). Previous studies have shown that beta-elemene has potent anti-tumor activity in several types of malignant tumors (7–10). More importantly, beta-elemene is relatively safe and has been widely used as an effective anticancer drug in humans (6, 11). Recent studies have shown that beta-elemene can reduce the malignancy of NSCLC cells by inhibiting their proliferation, migration and invasion (5, 8). Furthermore, beta-elemene can sensitize glioblastoma multiforme cells to gefitinib (12) and synergistically enhances its therapeutic effect on inhibiting stemness and progression of lung cancer by down-regulating EZH2 expression (13). However, the exact molecular mechanisms underlying the therapeutic action of beta-elemene remain largely indistinct, which may be an important obstacle for improving and refining the clinical application of beta-elemene.

Our previous study has indicated that chromosome 3 open reading frame 21 (C3orf21) can regulate the development of lung adenocarcinoma (14, 15). In this study, we tested the hypothesis that beta-elemene could modulate C3orf21 expression to attenuate the malignancy of NSCLC cells. The results indicated that beta-elemene treatment reduced the proliferation, wound healing and invasion of NSCLC cells by increasing C3orf21 expression. Such findings may provide new pharmacological evidence to explain why beta-elemene inhibits the malignancy of NSCLC.

## Materials and Methods

### Beta-Elemene

**Figure 1:**
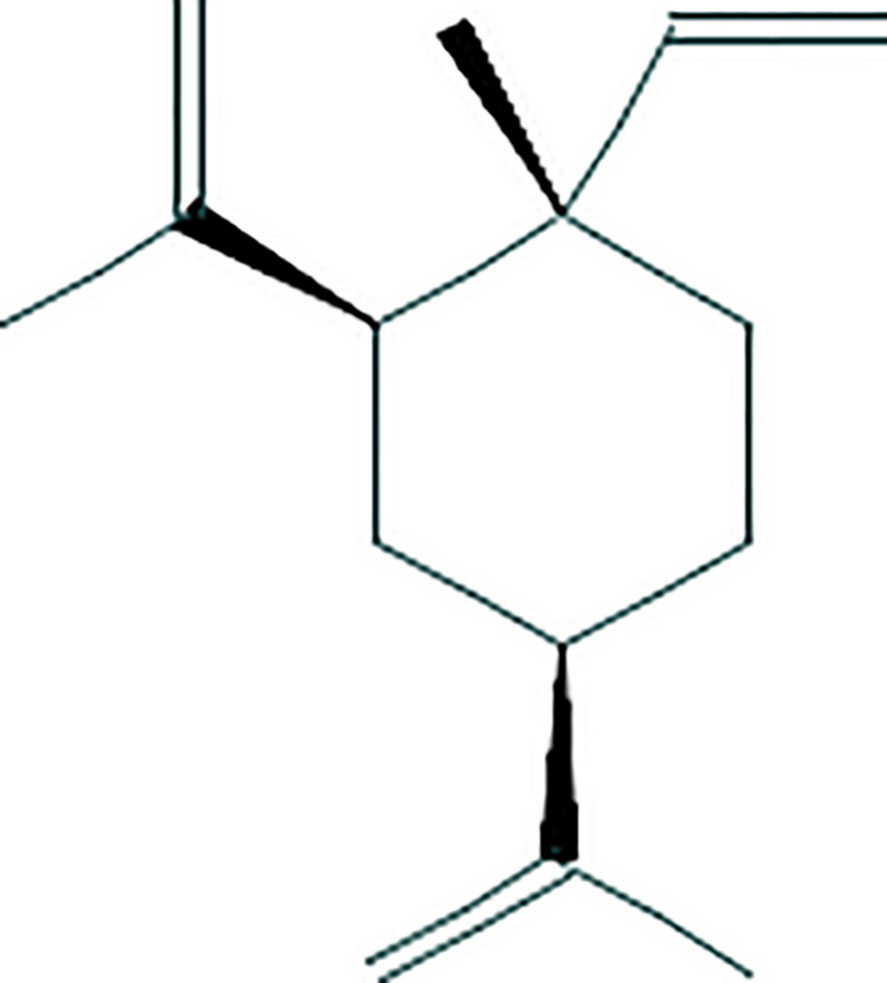
The structure of beta-elemene.

Beta-elemene [1-methyl-1-vinyl-2,4-diisopropenyl-cyclohexane, C15H24, molecular weight of 204.35, **Figure 1** (16)] is a sesquiterpene compound extracted from the dried rhizome of *Rhizoma curcumae*. The beta-elemene injection (xx% of beta-elemene, National Medical Product Administration approval Number: Chinese medicine H10960114) was produced by Dalian Huali Jingang Pharmaceutical (Dalian, China).

### Bioinformatic Analysis of Beta-Elemene-Associated Genes and Proteins

We searched the potential targets of beta-elemene using the BATMAN-TCM (http://bionet.ncpsb.org/batman-tcm/) database and STITCH (http://http://stitch.embl.de) database to predict the beta-elemene-associated genes and proteins. The BATMAN-TCM is an online bioinformatics analysis tool specially designed for studying the molecular mechanisms of traditional Chinese medicine, and is based on traditional Chinese medicine ingredients’ target prediction (17). The STITCH also is online Search Tool for Interacting Chemicals, which integrates these disparate data sources for 430,000 chemicals into a single, easy-to-use resource. The user can get a quick overview of the potential effects of the chemical on its interaction partners by STITCH database (18).

### Cells

We obtained human NSCLC A549, PC-9, NCI-H1975, MSTO-211H and NCI-H226 cells from Shanghai Institutes for Biological Sciences of Chinese Academy of Sciences. We identified them by STR. We grew them in RMPI1640 medium (Invitrogen) containing 10% fetal bovine serum (FBS, Gibco) in at 37°C 5% CO_2_. We treated the cells with, or without, the indicated concentrations of beta-elemene (Dalian Huali Jingang Pharmaceutical, Dalian, China) for varying time periods.

### Lentivirus Transduction

We cloned the DNA fragments for the expression of control and specific shRNAs (**Table 1**, Sangon Biotech, Shanghai, China) or the cDNA for C3orf21 expression into pLKO-ZSG-Puro, together with pCMV△R8.92 and pVSVG-I, to generate different types of lentiviruses in 293T cells. Subsequently, we transduced A549 cells with control or lentivirus for the expression of C3orf21-specific shRNA (Addgene) at multiplicity of infection of 4 in the presence of 8 mg/ml hexadimethrine bromide (Sigma). Similarly, we transduced PC-9 cells with control or lentivirus for the over-expression of C3orf21. Three days later, we treated the cells with puromycin (500 ng/ml) or blasticidin (10 mg/ml) to generate shCon, sh-C3orf21 stably silencing A549 and control, C3orf21 over-expressing PC-9 cells, respectively. We tested the efficacy of C3orf21 silencing or over-expression by fluorescent microscopy and Western blot assays.

**Table 1:** The sequences of primers and their applications.

Primer	Sequences (5’-3’)	Application
**C3rof21- F**	ATGTTGCTGTGCTGACGGATA	qRT-PCR
**C3rof21- R**	GGAGTCACTGTAGTAGGTTCCC	qRT-PCR
**C3rof21-F**		Overexpression
**C3rof21-R**		Overexpression
**shC3orf21**		Knockdown
**cyclinD1-F**	TCTGTTCCTCGCAGACCTCCAGCA	qRT-PCR
**cyclinD1-R**	CCGTCCATGCGGAAGATC	qRT-PCR
**cMyc-F**	CAGCTGCTTAGACGCTGGATT	qRT-PCR
**cMyc-R**	GTAGAAATACGGCTGCACCGA	qRT-PCR
**COX2-F**	AGATCATCTCTGCCTGAGTATCTT	qRT-PCR
**COX2-R**	TTCAAATGAGATTGTGGGAAAATTGCT	qRT-PCR
**MMP-2-F**	GGCCCTGTCACTCCTGAGAT	qRT-PCR
**MMP-2-R**	GGCATCCAGGTTATCGGGGA	qRT-PCR
**MMP-9-F**	AGGCCTCTACAGAGTCTTTG	qRT-PCR
**MMP-9-R**	CAGTCCAACAAGAAAGGACG	qRT-PCR
**VEGF-F**	GAAGTGGTGAAGTTCATGGATGTC	qRT-PCR
**VEGF-R**	CGATCGTTCTGTATCAGTCTTTCC	qRT-PCR
**PTEN-F**	CCGTTACCTGTGTGTGGTGATATC	qRT-PCR
**PTEN-R**	GAATGTATTTACCCAAAAGTGAAACATT	qRT-PCR
**GAPDH-assay-F**	CGGATTTGGTCGTATTG	qRT-PCR
**GAPDH-assay-R**	GAAGATGGTGATGGGATT	qRT-PCR

### Quantitative Real-Time-PCR (qRT-PCR)

We extracted total RNA from the different groups of cells using TRIzol (Invitrogen) and after qualification and quantification using an Agilent Bioanalyzer 2100 (Agilent Technologies), we reversely transcribed RNA samples into cDNAs using an iScript cDNA Synthesis Kit (BIO-RAD). Subsequently, we quantified the relative levels of mRNA transcripts by qRT-PCR using SYBR Premix ExTaqTM II kit (TaKaRa) and specific primers (**Table 1**). We performed RT-PCR in triplicate at 94°C for 10 s, and 40 cycles of 94°C for 5 s, 52°C for 30 s, 72°C for 15 s. We analyzed the data using the 2^−ΔΔCt^ method.

### CCK-8 Cell Assay

We determined the impact of beta-elemene on the proliferation of NSCLC cells by CCK-8 assay (Beyotime Shanghai China). Briefly, the cells (2–3.5 × 10^3^/well) from each group were grown in 96-well plates and treated in triplicate with beta-elemene at different concentrations for 24 h. Their proliferation was quantified with 10 μl/well of CCK-8 at 450 nm.

### Wound Healing Assay

The different groups of cells (2 × 10^5^ cells/well) were cultured in 6-well plate up to 80–90% confluence. The cell monolayer was wounded with a sterile micropipet tip and treated with, or without, the indicated doses of beta-elemene in serum-free medium. The monolayer of cells was photoimaged before and 24 h after beta-elemene treatment under an Olympus CKX-41 inverted microscope. The migrated areas of cancer cells were measured by Image J software.

### Transwell Invasion Assay

We examined whether beta-elemene could modulate NSCLC cell invasion by transwell invasion assay using the BioCoat Matrigel Invasion Chamber (Corning), according to the manufacturer’s recommended instructions. In brief, the different groups of cells (4 × 10^3^ cells/well) were cultured in triplicate in the upper chamber that had been coated with Matrigel (REF 3422, Corning). The bottom chambers were filled with 10% FBS medium. After 48-h culture, the invaded cells on the bottom surface of the upper chamber membranes were fixed and stained with crystal violet, followed by photoimaged. The invaded cells in five fields of each membrane were counted.

### Western Blot

We quantified the relative levels of interested proteins to the control GAPDH by Western blot. Briefly, we harvested each group of cells and lysed them in cell lysis buffer (Beyotime) containing a cocktail of protease inhibitors (Rocha). After qualification and quantification, we analyzed the cell lysates (30 µg/lane) by SDS-PAGE, and transferred to polyvinyl dene fluoride membranes (Millipore). After blocked, we incubated the membranes with anti-C3orf21 (20913-1-AP, Proteintech), anti-Notch1 (68309T), anti-GAPDH (2118S, Cell Signaling Technology) and detected the bound antibodies with HRP-conjugated second antibodies, followed visualizing using the enhanced chemiluminescence (Pierce, Rockford, USA). We quantified the data using Image J.

### Statistical Analysis

Data are present as mean ± SD. The difference between two groups was analyzed by Student’s T test, and the difference among multiple groups was analyzed by Two-way ANOVA using the GraphPad Prism 5.0 software. Statistical significance was defined when a P-value of ≤0.05.

## Results

### Bioinformatic Analysis of Beta-Elemene Targeting Genes and Proteins

**Figure 2:**
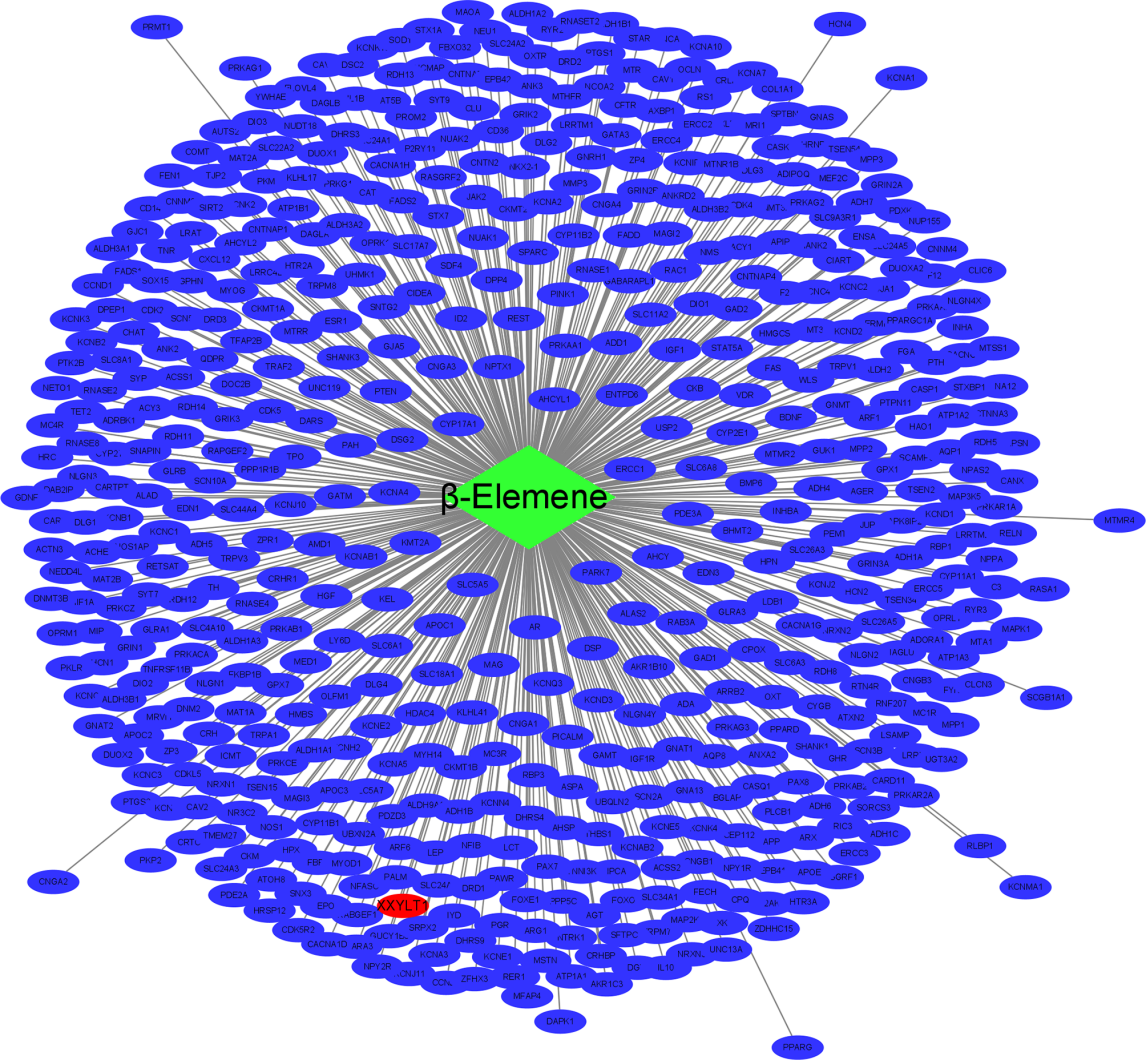
The network of beta-elemene with the potential targeted genes. green rhombus represents beta-elemene; blue ellipse represent the targeted genes of beta-elemene; red ellipse represent the XXYLT1.

**Figure 3:**
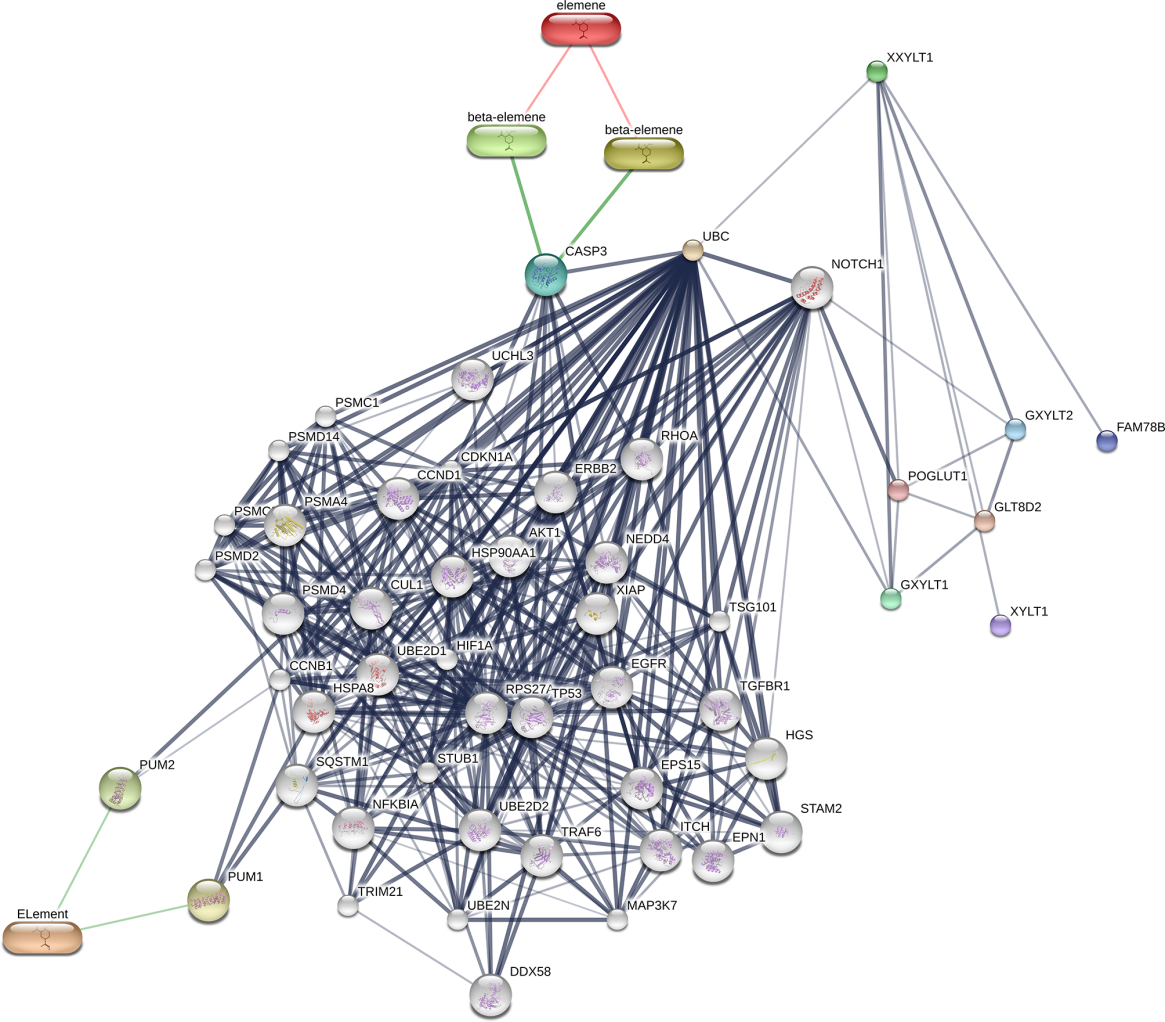
A network of beta-elemene with the potential interacted proteins. Gray lines indicate protein-protein interactions; Green lines indicate compound–protein interactions.

To search the potential genes and proteins associated with beta-elememe, we performed bioinformatics analysis using beta-elemene (6918391, PubChem CID) as a key word to search the BATMAN-TCM database. With a cutoff score ≥5, we obtained 522 genes associated with beta-elemene (**Figure 2**). To further search the beta-elemene-associated proteins, we used the STITCH database and found that fifty proteins were associated with beta-elemene, including XXYLT1, Notch and others (**Figure 3**).

### Beta-Elemene Enhances C3orf21 Expression in NSCLC Cells

**Figure 4:**
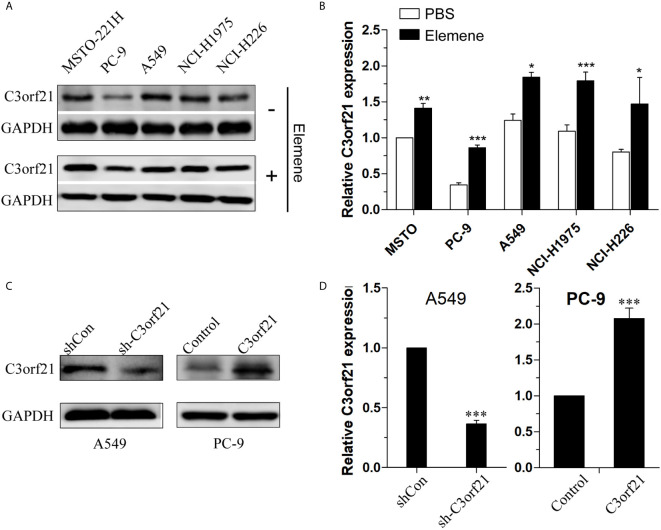
C3orf21 expression in NSCLC cells. **(A)** Western blot analysis of the relative levels of C3orf21 expression in the indicated NSCLC cells. **(B)** Beta-elemene enhances C3orf21 expression in NSCLC cells. **(C, D)** Generation of C3orf21 stable silencing A549 cells and C3orf21 stable over-expressing PC-9 cells. Data are representative images or expressed as the mean ± SD of each group from three biological experiments. *P < 0.05, **P < 0.01 and ***P < 0.001 vs. the control.

C3orf21 has shown to act as a tumor suppressor of several types of malignant tumors. To understand the antitumor effect of beta-elemene, we tested whether beta-elemene treatment could modulate C3orf21 expression in several NSCLC cell lines. Western blot analysis indicated that C3orf21 expression varied in the different NSCLC cell lines (**Figure 4A**) and treatment with beta-elemene (10 μg/ml) for 24 h significantly up-regulated C3orf21 expression in these NSCLC cells (p < 0.05—p < 0.001, **Figure 4B**). Given that A549 cells expressed relatively higher levels of C3orf21 while PC-9 expressed lower levels of C3orf21, we generated C3orf21 stably silenced A549 and C3orf21 over-expressed PC-9 cells (**Figures 4C, D**) and these cell lines were valuable for determining the role of altered C3orf21 expression in regulating the beta-elemene-mediated anti-NSCLC effects.

### Beta-Elemene Inhibits the Proliferation of NSCLC Cells, Dependent on C3orf21 Expression

**Figure 5:**
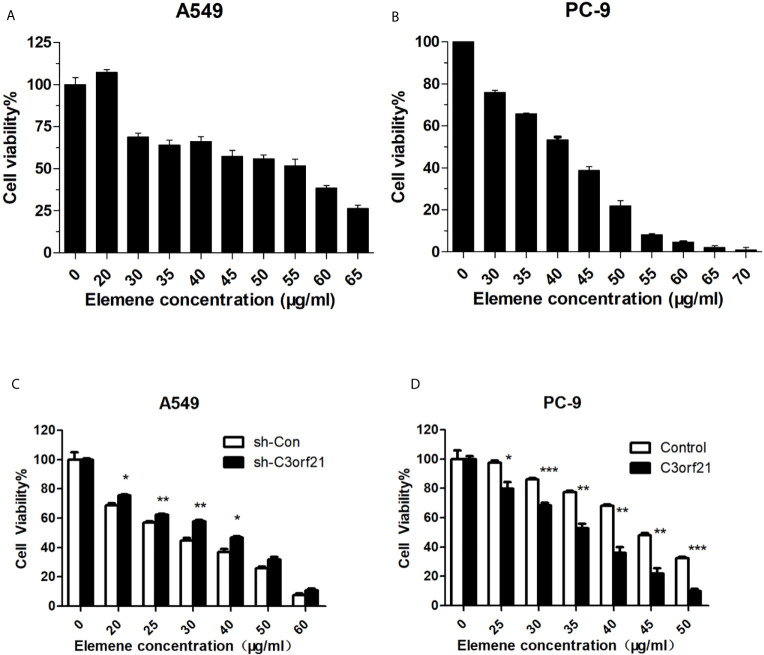
Beta-elemene inhibits the proliferation of NSCLC cells. A549, PC-9, A549-shNC and A549-shC3orf21 cells were treated with, or without, the indicated concentrations of beta-elemene for 24 h and the cell proliferation was determined by CCK-8 assays. Data are expressed as the mean ± SD of each dose group from three biological experiments. **(A, B)** The proliferation of A549 cells. **(B)** The proliferation of PC-9 cells. **(C)** The proliferation of A549-shNC and A549-shC3orf21 cells. **(D)** The proliferation of PC-9-NC and PC-9-C3orf21 over-expression. *P < 0.05, **P < 0.01 and ***P < 0.001 vs. the control.

To investigate how altered C3orf21 expression modulated the anti-NSCLC effect of beta-elemene, we first tested the impact of beta-elemene on A549 and PC-9 cell viability by CCK-8 assays. We found that treatment with beta-elemene significantly decreased A549 and PC-9 cell viability in a dose-dependent manner (**Figures 5A, B**). Furthermore, C3orf21 silencing dramatically mitigated the inhibitory effects of beta-elemene on the proliferation of A549 cells while C3orf21 over-expression remarkably enhanced its inhibitory effects on the proliferation of PC-9 cells (**Figures 5C, D**). These data indicated that beta-elemene attenuated the proliferation of NSCLC cells, which was regulated by C3orf21 expression.

### Beta-Elemene Attenuates the Wound Healing of NSCLC Cells Dependent on C3orf21 Expression

**Figure 6:**
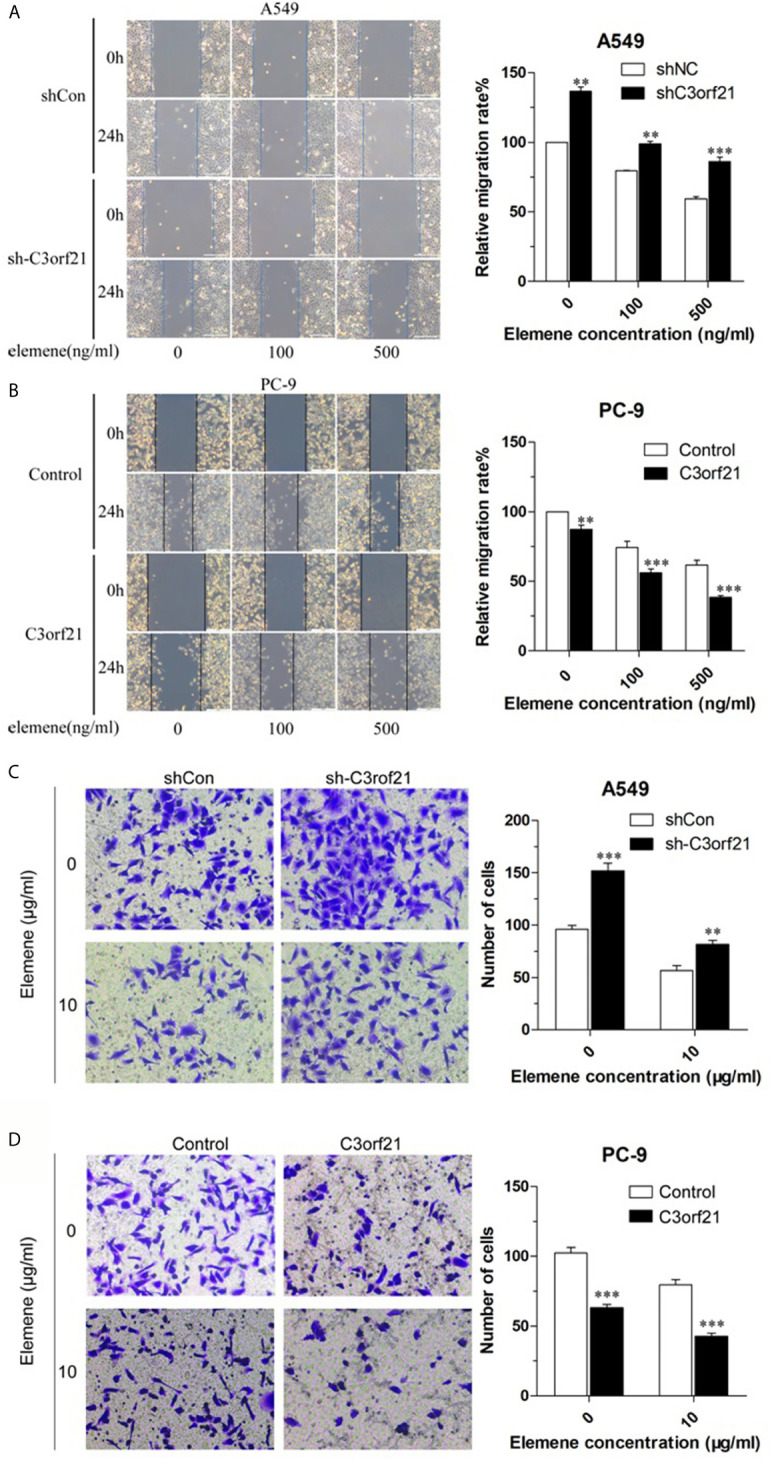
Beta-elemene attenuates the wound healing and invasion of NSCLC cells. **(A)** The wound healing of A549 cells. **(B)** The wound healing of PC-9 cells. **(C)** The invasion of A549 cells. **(D)** The invasion of PC-9 cells. Data are representative images or expressed as the mean ± SD of each group from three biological experiments. **P < 0.01 and ***P < 0.001 vs. the control.

To further explore how beta-elemene modulated the malignancy of NSCLC cells, we tested whether altered C3orf21 expression could change the effect of beta-elemene on the wound healing of shNC-A549, sh-C3orf21-A549, control-PC-9 and C3orf21-PC-9 cells. While C3orf21 silencing significantly increased the wound healing of shNC-A549 cells treatment with beta-elemene significantly decreased their wound healing and the inhibitory effects tended to be dose-dependent (**Figure 6A**). Similarly, beta-elemene treatment also significantly attenuated the wound healing of C3orf21-silenced A549 cells, but the wound healing effects in the C3orf21-silenced A549 cells were significantly stronger relative to their corresponding shNC-A549 cells. Furthermore, beta-elemene treatment also significantly minimized the wound healing of control-PC-9 cells and further decreased it in the C3orf21-over-expressed PC-9 cells (**Figure 6B**). A similar pattern of beta-elemene treatment on the invasion of shNC-A549, sh-C3orf21-A549, control-PC-9 and C3orf21-PC-9 cells was observed by transwell invasion assays (**Figures 6C, D**). Collectively, C3orf21 silencing mitigated the inhibition of beta-elemene on the wound healing and invasion of NSCLC while C3orf21 over-expression enhanced its inhibitory effects in NSCLC cells.

### Beta-Elemene Modulates the Expression of Several Regulators and Signal Events in NSCLC Cells, Dependent on C3orf21 Expression

**Figure 7:**
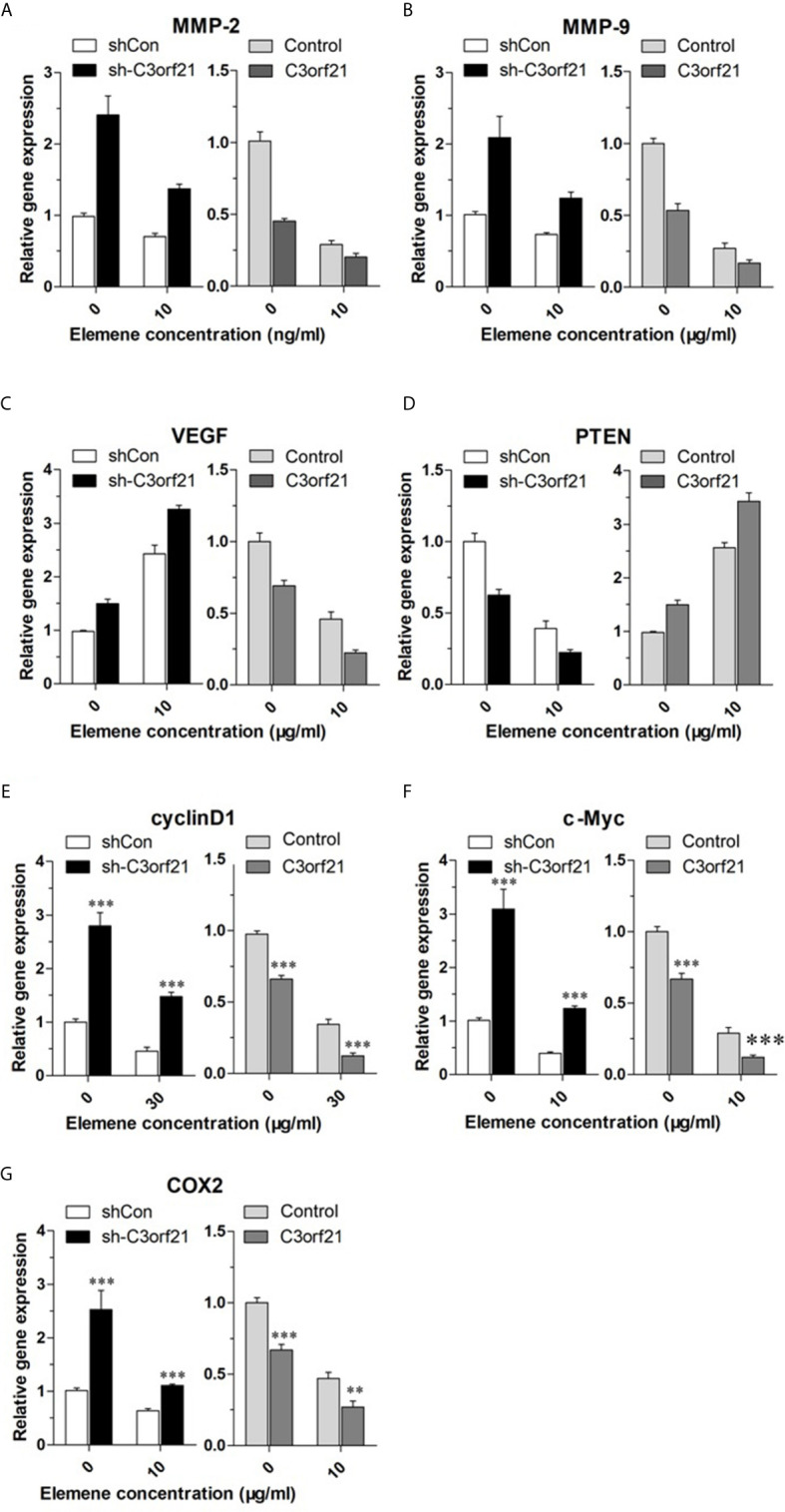
Beta-elemene modulates the expression of several regulators and signal events in NSCLC cells, dependent on C3orf21 expression. The indicated NSCLC cells were treated with, or without, beta-elemene for 24 h and the relative levels of MMP-2 **(A)**, MMP-9 **(B)**, VEGF **(C)**, PTEN **(D)** Cyclin **(E)**, C-Myc **(F)**, and COX2 **(G)** in the indicated NSCLC cells were quantified by RT-PCR. Data are present as mean ± SD of each group from at least three biological experiments. **P < 0.01 and ***P < 0.001 vs. the control.

**Figure 8:**
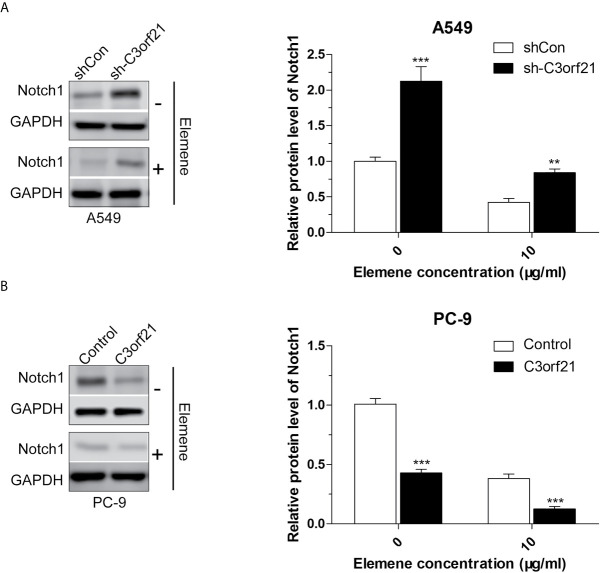
Beta-elemene decreases C3orf21 protein expression in NSCLC cells. The indicated NSCLC cells were treated with, or without, beta-elemene for 24 and their relative levels of Notch1 protein expression **(A, B)** were quantified by Western blot. Data are representative images or present as mean ± SD of each group from at least three biological experiments. **P < 0.01 and ***P < 0.001 vs. the control.

To further understand the pharmacological action of beta-elemene in attenuating the malignancy of NSCLC cells, we examined how beta-elemene treatment could modulate the levels of Cyclin D1, c-Myc, COX2, MMP2, MMP9, VEGF and PTEN as well as Notch1 mRNA transcripts and protein expression in different groups of NSCLC cells. As shown in **Figure 7**, C3orf21 silencing significantly increased the relative levels of cyclin D1, c-Myc, Cox2, MMP2, MMP9, VEGF, but decreased PTEN mRNA transcripts in A549 cells. Conversely, C3orf21 over-expression had opposite effects on their expression in PC-9 cells, relative to their controls. Treatment with beta-elemene significantly decreased Cyclin D1, c-Myc, COX2, MMP2, MMP9, PTEN, but increased VEGF mRNA transcripts in both control and C3orf21-silenced A549 cells, relative to that of untreated cells. In addition, beta-elemene treatment also deceased Cyclin D1, c-Myc, COX2, MMP2, MMP9 and VEGF, but increased PTEN mRNA transcripts in both control and C3orf21-over-expressed PC-9 cells, compared with that in their untreated cells. Western blot analysis indicated that C3orf21 silencing up-regulated Notch1 expression in A549 cells while C3orf21 over-expression down-regulated Notch1 expression in PC-9 cells (**Figure 8**). Treatment with beta-elemene significantly reduced Notch1 expression in all groups of cells, particularly in the C3orf21-over-expressed PC-9 cells. Together, such data indicated that beta-elemene treatment modulated the expression of several key regulators and signal events in NSCLC cells, dependent on C3orf21 expression.

## Discussion

NSCLC is the most prevalent malignancy, and current treatments for NSCLC include surgical resection, target therapies and chemotherapy, which have side effects. Hence, discovery of new safe and therapeutic reagents is urgently needed. Previous studies have shown that beta-elemene has potent antitumor activity and can minimize the drug resistance of some types of tumor cells (19–21). In this study, we found that beta-elemene significantly enhanced C3orf21 expression in NSCLC cells. Beta-elemene treatment dramatically decreased the proliferation, would healing and invasion of NSCLC cells, which were mitigated by C3orf21 silencing, but enhanced by C3orf21 over-expression in vitro. The significantly decreased proliferation, wound healing and invasion of NSCLC cells indicated that beta-elemene remarkably attenuated the malignancy of NSCLC cells by up-regulating C3orf21 expression. Such novel findings extended previous observations and support the notion that C3orf21 acts as a tumor suppressor in inhibiting the development and progression of malignant tumors. Moreover, C3orf21 over-expression further enhanced the therapeutic effect of beta-elemene, which suggests that C3orf21 may be a new therapeutic target for intervention of NSCLC.

To understand the pharmacological action of beta-elemene, we tested whether beta-elemene treatment could modulate the expression of key regulators for cell behaviors and signal events in NSCLC cells. We found that C3orf21 silencing remarkably increased the relative levels of Cyclin D1, c-Myc, Cox2, MMP2, MMP9, VEGF, besides Notch1, but decreased PTEN expression while C3orf21 over-expression displayed opposite effects in NSCLC cells. Such results extended previous observations that C3orf21 inhibits the Notch signaling (22). It is well known that Cyclin D1, c-Myc and Cox2 can promote the proliferation of tumor cells (23) while PTEN can suppress the PI3K/AKT/mTOR signaling (24). Furthermore, VEGF is an important angiogenic factor and MMP2/9 are crucial for tumor cell motility (25). In addition, decreased levels of C3orf21 expression is related to a poor prognosis in patients with lung cancer (26). Functionally, C3orf21 can prolong the O-linked xylose-glucose (14). The inhibitory effect of C3orf21 on the Notch signaling is likely associated with the differential O-linked glycosylation of its extracellular domain (NECD) (27, 28). More importantly, we found that beta-elemene treatment significantly decreased cyclin D1, c-Myc, Cox2, MMP2/9 expression, which were attenuated by C3orf21 silencing and enhanced by C3orf21 over-expression in NSCLC cells. Interestingly, beta-elemene treatment significantly increased VEGF expression, but decreased PTEN expression in C3orf21-sielnced A549 cells. The same treatment had opposite effects on the VEGF and PTEN expression in the C3orf21-over-expressed PC-9 cells. It is possible that C3orf21 may through the similar mechanisms, regulate the expression of other molecules tested. We are interested in further investigating how C3orf21 regulates the expression and functions of these regulators and signaling during the development and progression of NSCLC.

Collectively, our results revealed that beta-elemene treatment dramatically enhanced C3orf21 expression in NSCLC cells. Beta-elemene treatment significantly reduced the malignancy of NSCLC cells, which were attenuated by C3orf21 silencing, but enhanced by C3orf21 over-expression. Similarly, we found that beta-elemene treatment significantly altered the expression of key regulators of malignant behaviors and signal events, which were modulated by altered C3orf21 expression in NSCLC cells. Thus, our findings may provide new insights into the pharmacological action of beta-elemene in inhibiting the development and progression of NSCLC.

## Data Availability Statement

The raw data supporting the conclusions of this article will be made available by the authors, without undue reservation.

## Author Contributions

HC and YZ were responsible for study design, performing experiments, statistical analysis, and manuscript writing. LR and YW collected the data and wrote the manuscript. All authors contributed to the article and approved the submitted version.

## Funding

This study was supported by grants from the Zhejiang Provincial Medical and Health Scientific and Technical Foundation (No.2017171306, 2018KY296 and 2020KY470).

## Conflict of Interest

The authors declare that the research was conducted in the absence of any commercial or financial relationships that could be construed as a potential conflict of interest.
